# Biofilms in chronic diabetic foot ulcers—a study of 2 cases

**DOI:** 10.3109/17453674.2011.581265

**Published:** 2011-07-08

**Authors:** Daniëlle Neut, Elvira JA Tijdens-Creusen, Sjoerd K Bulstra, Henny C van der Mei, Henk J Busscher

**Affiliations:** ^1^Departments of Biomedical Engineering; ^2^Orthopaedic Surgery, University Medical Center Groningen and University of Groningen, Groningen, the Netherlands

Among the 55 million people in Europe diagnosed as having diabetes mellitus (www.diabetesatlas.org), the lifetime risk of developing a foot complication—often infected wounds—could be as high as 25% (Boulton et al. 2005). Many of the patients with severe infections will require amputation within the foot or above the ankle. Approximately 85% of lower-limb amputations in patients with diabetes are preceded by infected foot ulceration (Adler et al. 1999).

Polymicrobial infections predominate in severe diabetic foot infections (Dowd et al. 2008a) and the medical community is now beginning to realize that the diversity of bacterial populations in chronic wounds (Dowd et al. 2008b, James et al. 2008) may be an important contributor to the chronicity of wounds, such as diabetic foot ulcers. In addition, another obstacle to the healing of chronic wounds is the biofilm mode of growth of the infecting organisms (Wolcott and Rhoads 2008). By definition, biofilms are microbial populations that are attached to a surface, or to the surfaces of other organisms, and encase themselves in hydrated extracellular polymeric substance (EPS), which is also referred to as “slime”. The chemical and physical properties of EPS can vary, but it is mainly composed of polysaccharides. EPS is also associated with other macromolecules such as proteins, DNA, lipids, and even humic substances (Nielsen et al. 1996, Tsuneda et al. 2003).

Biofilm-related diseases are usually persistent infections that develop slowly. They appear to be rarely cleared by the host immune system and are highly resistant to antimicrobial therapy (Stewart and Costerton 2001). For example, antibiotic resistance in biofilm bacteria of up to 1,000 times that of planktonic bacteria has been extensively documented in experiments in vitro (Stewart and Costerton 2001). Infected diabetic foot ulcers share these characteristics, and it has been hypothesized that biofilms may play a role in these infections (Davis et al. 2006, Dowd et al. 2008a). We evaluated debrided soft tissue from infected diabetic foot ulcers by confocal laser scanning microscopy (CLSM). Part of the debrided tissue was also analyzed using culture methods to evaluate bacterial diversity of the biofilms.

## Case 1

A 58-year-old man with type-2 diabetes since 2000 presented with a non-healing ulcer on the top of the left great toe. He was initially treated with multiple courses of antibiotics and a total contact cast followed by a rigid orthopedic shoe and multiple debridements at the outdoor clinic. After 7 months, we decided to perform an amputation of the toe because of the non-healing tendency of the ulcer. His HbA1c at that time was 7.2.

## Case 2

A 69-year-old man with type-1 diabetes presented at the orthopedic outdoor patient clinic with a non-healing ulcer on the medial, plantair site of the left foot at the level of the os naviculare. He was treated with oral antibiotics (ciprofloxacin 2 × 500 mg and clindamycin 3 × 600 mg) for 6 weeks and a total contact cast and multiple debridements at the outpatient clinic. After 6 months, we decided to perform a clinical debridement in the operating theater due to insufficient healing of the ulcer.

Soft tissue samples were collected with sterile tools from these 2 patients when undergoing deep debridement in the operating theater. Once the debridement was complete, material debrided from the wound was prepared for CLSM examination and microbiological culture. Both patients gave their oral consent.

Tissue samples were stained with LIVE/DEAD *Bac*light viability stain (Molecular Probes Europe BV, Leiden, the Netherlands) containing SYTO 9 dye (fluorescent green) and propidium iodide (fluorescent red) to differentiate between living and dead bacteria, respectively. In addition, samples were stained with calcofluor white (0.1 mM; fluorescent blue), a polysaccharide-binding dye used to visualize EPS. Samples were examined using a Leica TCS-SP2 CSLM microscope (Leica Microsystems Heidelberg GmbH, Heidelberg, Germany).

## Results and Discussion

**Figure 1.**
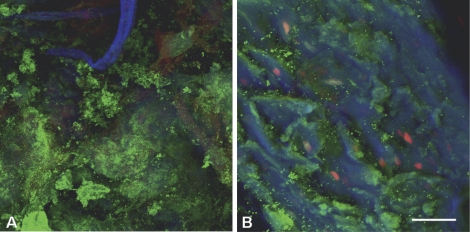
CLSM images of biofilm on soft tissue from patient 1. Overlay projection (includes all the slices in an image stack) of the biofilm at the center of the ulcer base (A) and at the edge of the ulcer base (B). Bar represents 75 µm. CLSM examination revealed the presence of bacteria ranging from single cells to large aggregates of grape-like clusters (panel A). The bacteria in these clusters were viable, as they appeared fluorescent green after LIVE/DEAD *Bac*light viability stain. Calcofluor white (blue) stained the EPS excreted by the bacteria (panel B). Host nuclei and fibrous material stained red with propidium iodide (panels A and B).

**Figure 2.**
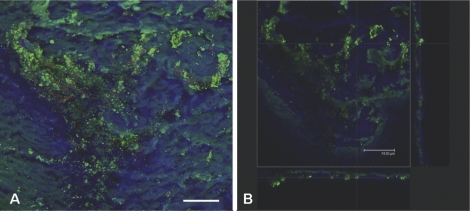
CLSM images of biofilm on soft tissue from patient 2. Overlay projection of the biofilm (A) and a projected side-view (B), meaning the biofilm is visualized from the top and from the side (XZ-plane and YZ-plane, respectively). Bar represents 75 µm. CLSM examination showed that the biofilm distribution was patchy (panel A), with some sites containing large clusters of bacteria and other regions showing hardly any evidence of infection. The bacteria were frequently embedded within a self-produced matrix of EPS (panel B). In addition, the infected ulcer was quite superficial, as can be concluded from the biofilm thickness shown in panel B.

The clinical progression of both patients corresponded to the behavior expected when there is a chronic underlying biofilm infection that is resistant to standard antibiotic therapy. CLSM examination revealed the presence of densely aggregated colonies of bacteria often surrounded by EPS and host-cell debris (Figures 1 and 2). The distribution was patchy, the presence of bacteria ranging from single cells to large aggregates of grape-like clusters (Figure 1A and Figure 2A). The bacteria in these clusters were viable as they turned fluorescent green after staining with LIVE/DEAD Baclight viability stain. Calcofluor white stained the EPS excreted by the bacteria fluorescent blue (Figure 1B and Figure 2B). These morphological observations are characteristic of biofilms and they provide evidence that biofilms can be present in chronic infected diabetic foot ulcers. In addition, the infected ulcer was quite superficial—as can be concluded from the thickness of the biofilm shown in Figure 2B.

Standard microbiological culture revealed a biofilm species composition in patient 1 of *Staphylococcus aureus* (MRSA-negative), *Proteus vulgaris, Enterococcus avium, *and *Enterococcus faecalis.* Standard culture revealed a biofilm species composition in patient 2 of *Staphylococcus aureus* (MRSA-negative) and *Enterobacter cloacae.*

The demonstration of living bacteria on tissue from an ulcer base suggests that a well-developed mature biofilm can adhere to soft tissue. In orthopedic infections, biofilms are usually discussed in the context of growth on a foreign body (Costerton 2005) or dead bone. Our data show that infected foot ulcers from diabetic patients may also act as a reservoir for pathogenic biofilms. It has been speculated for several years that bacteria that colonize chronic wounds exist as biofilm communities (Serralta et al. 2001, Percival and Bowler 2004). Chronic wound infections share two important characteristics with other biofilm diseases: persistent infection that is not cleared by the host immune system and resistance to antimicrobial therapy. However, there has been very little direct evidence of biofilm involvement in chronic wound infections (Kirketerp-Moller et al. 2008). We have identified viable biofilms with CLSM-based visualization techniques on debrided tissue from diabetic patients with an infected ulcer, and these findings were confirmed by culture on agar plates. In both cases, *S. aureus* (MRSA-negative) was retrieved from the ulcer, while other bacteria were also present in each ulcer. To our knowledge, this is the first reported direct demonstration of bacterial infections associated with a diabetic foot ulcer that fulfill all of the established criteria for a biofilm infection (Hall-Stoodley et al. 2006, Stoodley et al. 2008).

Frequently, ulceration in the diabetic foot is partly caused by vascular insufficiency; because of the poor blood flow, antibiotics cannot easily get to the site of the infection. Plans for treatment may therefore include improvement of the blood circulation (arterial revascularization). However, we found that infected foot ulcers may involve biofilm infection and the role of biofilms in treating infected foot ulcers is not usually addressed. Bacteria within mature biofilms are highly resistant to many traditional antimicrobial therapies (Davis et al. 2006). For example, antibiotic resistance in biofilm bacteria of up to 1,000 times that of planktonic bacteria has been extensively documented in experiments in vitro (Stewart and Costerton 2001). Thus, both systemic and topical antibiotics alone are unable to eradicate biofilm infections. This suggests that if systemic antibiotics are used, they must be used together with topical antiseptic and anti-biofilm strategies. Currently, one of the most successful strategies for the management of biofilm infections is physical removal of the biofilm, such as frequent debridement of diabetic foot ulcers. This includes proper wound cleansing and scraping off of all dead tissue. In addition, future treatment plans should consider application of local antibiotic-delivery systems, as in infected joint replacements. With small cement beads or collagen fleeces, antibiotic concentrations at the site of infection can be achieved that are far higher than can be obtained after systemic application of the same antibiotic (Walenkamp 2001). As Pollard (2008) indicated in a commentary recently, biofilm-based wound care is “a significant shift in our whole approach to wound healing”.
